# MCC‐135 Exerts Antiepileptic and Neuroprotective Effects by Downregulating NCX1 Expression to Decrease Intracellular Calcium Overload in the Hippocampus

**DOI:** 10.1002/cns.70808

**Published:** 2026-03-02

**Authors:** Chaoning Liu, Min He, Rida Li, Shouhuan Zheng, Lanfeng Sun, Chi Gong, Hengchang Qi, Xinran Qin, Xiaohang Gan, Fang Wang, Yuan Wu

**Affiliations:** ^1^ Department of Neurology The First Affiliated Hospital of Guangxi Medical University Nanning Guangxi People's Republic of China

**Keywords:** calcium overload, epilepsy, glutamate release, MCC‐135, NCX1

## Abstract

**Background:**

Approximately 30% of epilepsy patients still develop drug resistance after standard antiepileptic treatment. Therefore, there is an urgent need to identify new drug targets to improve seizure control. Previous studies have shown that NCX1 can regulate the intracellular Ca^2+^ levels in astrocytes and neurons, which are closely associated with epilepsy. MCC‐135 has shown potential as an antiseizure medication due to its ability to downregulate NCX and reduce intracellular calcium overload; however, its role and mechanism in epilepsy remain unclear.

**Methods:**

This study employed single‐cell analysis and molecular docking to identify the potential molecular targets of MCC‐135 in treating epilepsy. Additionally, we used a KA‐induced epileptic mouse model to validate these molecular levels and the therapeutic effects and mechanisms of MCC‐135.

**Results:**

Relative to controls, NCX1 expression was significantly upregulated in the hippocampus of KA‐induced epileptic mice. Immunofluorescence staining revealed that NCX1 was co‐localized with both astrocytes and neurons. MCC‐135 treatment significantly prolonged the seizure latency in KA‐induced epileptic mice and alleviated hippocampal neuronal damage. Furthermore, MCC‐135 effectively reduced NCX1 expression, alleviated intracellular calcium overload, and downregulated glutamate levels in the epileptic mice.

**Conclusion:**

MCC‐135 exerts neuroprotective and antiepileptic effects by downregulating NCX1 expression, thereby alleviating calcium overload and reducing glutamate levels in the hippocampus. We are the first to propose the role and mechanism of MCC‐135 in epilepsy treatment, providing novel insights into its potential as a therapeutic agent for epilepsy.

## Graphical abstract

MCC‐135 treatment significantly prolonged the seizure latency and alleviated hippocampal neuronal damage in KA‐induced epileptic mice. Furthermore, MCC‐135 reduced NCX1 levels, alleviated intracellular calcium overload, and downregulated glutamate levels in the epileptic mice. We are the first to propose the role and mechanism of MCC‐135 in epilepsy treatment.

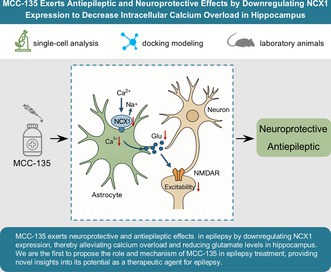

## 1 Introduction

Epilepsy is one of the most common neurological disorders worldwide, affecting over 70 million people of all age groups [1]. Despite the availability of more than 30 antiseizure medications (ASMs), approximately 30% of epilepsy patients exhibit resistance to standard ASM treatments, resulting in uncontrolled seizures and significantly impairing their quality of life [2]. Therefore, there is an urgent need to identify alternative drug targets and novel therapeutic approaches to improve seizure control in epilepsy.

The sodium/calcium exchanger (NCX) primarily mediates the transport of sodium ions (Na^+^) and calcium ions (Ca^2+^) across the cell membrane, thereby regulating their levels. NCX is a member of the solute carrier family 8 (SLC8), and three major isoforms have been identified in mammals: NCX1, encoded by the *SLC8A1* gene; NCX2, encoded by the *SLC8A2* gene; and NCX3, encoded by the *SLC8A3* gene. NCX1 is the most widely expressed member of the SLC8 family, found in neurons, astrocytes (Ast), epithelial cells, and other cell types. At the post‐transcriptional level, at least 17 isoforms of NCX1 are generated through selective splicing of the *SLC8A1* transcript [3]. In the hippocampus, NCX1 is significantly expressed in dendrites and dendritic spines at asymmetric axon terminal contacts, expressed in the cytoplasmic form and located at the distal protrusions surrounding excitatory synapses [4]. Glial NCX1 is predominantly expressed in astrocytic processes ensheathing excitatory synapses [5]. Thus, NCX1 is more likely to regulate the resting and transient levels of Na^+^ and Ca^2+^ on the postsynaptic membrane of excitatory synapses.

It has been confirmed that the Na^+^ influx in astrocytes generates Na^+^ transients, and the resulting calcium influx driven by the NCX1 reverse mode plays a key role in the dynamic coupling of calcium transients in both neurons and astrocytes [3]. The increase in intracellular calcium concentration ([Ca^2+^]_i_) in astrocytes is associated with the initiation and maintenance of focal epileptiform discharges [6]. Additionally, the increase in [Ca^2+^]_i_ in astrocytes can induce the release of glutamate (Glu) [7], raising its concentration in the synapse, leading to excitotoxicity and neuronal hyperexcitability, thereby triggering epilepsy [8]. Inhibition of the rise in astrocytic [Ca^2+^]_i_ can reduce epileptic‐like behavior in mice by 60% [9]. This process appears to rely on a feedback mechanism—an increase in [Ca^2+^]_i_ leads to Glu release by astrocytes, which in turn contributes to additional neurons becoming hyperactive [10]. Once the number of hyperactive neurons exceeds a critical threshold, epilepsy may occur. Therefore, the reverse mode of NCX1 may mediate epilepsy by increasing [Ca^2+^]_i_ and inducing Glu release in astrocytes.

MCC‐135 is 5‐methyl‐2‐[piperazine‐1‐yl] benzenesulfonic acid monohydrate. Its mechanism of action is to reduce intracellular calcium overload by downregulating NCX expression on the cell membrane or enhancing the ability of the sarcoplasmic reticulum to take up additional calcium [11]. However, the role and mechanism of MCC‐135 in epilepsy remain unclear. Under the pathological conditions of epilepsy, intracellular Ca^2+^ overload exacerbates membrane potential fluctuations, promoting excessive presynaptic Glu release, leading to abnormal neuronal synchronization, and then triggering seizures [12]. This process highlights the central role of calcium homeostasis disruption in the pathogenesis of epilepsy and provides a theoretical basis for targeting calcium signaling in antiepileptic therapy. Given that MCC‐135 can reduce intracellular calcium overload by downregulating NCX expression, further research into its potential in epilepsy is warranted.

In this study, we integrated bioinformatics with experimental methods to analyze the properties of MCC‐135. We first analyzed the influence of astrocytic *SLC8A1* (encoding NCX1) subpopulations on calcium‐related signaling pathways by using single‐cell RNA sequencing data. Then, we employed molecular docking technology to evaluate the potential interaction between the NCX1 protein and MCC‐135 and confirmed these results in an epilepsy mouse model. This study seeks to know if MCC‐135 can improve seizures by downregulating NCX1 expression, reducing intracellular calcium overload, and lowering Glu levels in epilepsy.

## 2 Materials and Methods

### 2.1 Data Collection

We accessed bulk RNA sequencing (RNA‐seq) data from the Gene Expression Omnibus (GEO) (www.ncbi.nlm.nih.gov/geo) for healthy control and epilepsy patients. The GSE33000 dataset (including 157 normal human brain tissue samples) and the GSE63808 dataset (including 129 brain tissue samples of epileptic patients) were used to screen genes with significantly different expression between the epileptic group and the normal group.

To investigate the transcriptomic changes in various cell types after the onset of epilepsy, we downloaded single‐cell/single‐nucleus RNA sequencing (sc/snRNA‐seq) datasets from healthy controls and epilepsy patients from the GEO database. After filtering out non‐human and non‐sc/snRNA‐seq data, we obtained 25 epilepsy patient samples and 32 healthy control samples. These include datasets from the GPL24676 platform: GSE160189 [13], GSE221849 [14], GSE129308 [15], GSE241521 [16], GSE157827 [17], GSE174367 [18], GSE178175 [19], and GSE213364 [20]. Additionally, data from the GPL20301 platform, including GSE140393 [21] and GSE163737 [22], were used.

### 2.2 Data Preprocessing and Quality Control

The scDatamerge application integrates the raw data from the 10X sc/snRNA‐seq pipeline or expression data in a data frame format and generates a single‐cell expression profile (Seurat object). The RunSeuratQC application performs quality control on single‐cell Seurat objects using the Seurat tool [23], specifically for doublets, dead cells, and mitochondrial genes. The default filtering thresholds are cells within the top and bottom 1% of feature (gene) counts for each patient, and mitochondrial content less than 10%. The RunSeuratNorm application normalizes the quality‐controlled sc/snRNA‐seq data by removing batch effects and performing data standardization.

### 2.3 Cell Clustering and Subpopulation Identification

The Seurat package was used to cluster the sc/snRNA‐seq data [23], and the Uniform Manifold Approximation and Projection (UMAP) package was employed to visualize the dimensional reduction and clustering identification [24]. Cell clusters were identified using markers from published studies in the CellMarker database, and the FindAllMarkers function was used to identify the specific expression genes for each cluster subpopulation [25], followed by annotation of the subpopulations for different cell types.

### 2.4 Functional Enrichment Within Cell Clusters

To investigate the biological functions of distinct cellular subpopulations, we employed the clusterProfiler package in R to perform Gene Ontology (GO) and Kyoto Encyclopedia of Genes and Genomes (KEGG) pathway enrichment analyses based on the marker genes of each subpopulation [25, 26]. Enrichment terms with an adjusted *p*‐value < 0.05 were considered statistically significant.

### 2.5 Animals and Grouping

Male C57BL/6 mice (approximately 20 g, 6 weeks old) were obtained from the Animal Experimental Center of Guangxi Medical University. All mice were housed under controlled humidity and temperature in well‐ventilated rooms, maintained on a 12 h light/dark cycle, with free access to food and water. Mice were acclimatized for seven days prior to experimentation. All procedures were conducted in accordance with the NIH *Guide for the Care and Use of Laboratory Animals*. The study protocol was reviewed and approved by the Animal Ethics Committee of Guangxi Medical University.

**Experiment 1**: Twelve mice were randomly and equally assigned to the sham‐operated group (sham) and the epilepsy group. Mice in the epilepsy group received a unilateral intrahippocampal injection of kainic acid (KA) into the right hippocampus to induce seizures, while sham mice received an equal volume of physiological saline at the same site.

**Experiment 2**: To evaluate whether MCC‐135 modulates epilepsy by downregulating NCX1 expression, mice were pretreated with intraperitoneal injections of MCC‐135 prior to induction of the KA epilepsy model. To determine the optimal therapeutic dose, mice were randomly assigned to the following groups: the sham‐operated group (sham^+^Vehicle), the model group (KA^+^Vehicle), and low‐, medium‐, or high‐dose treatment groups (KA^+^MCC‐135). Mice in the low‐, medium‐, and high‐dose groups received intraperitoneal injections of MCC‐135 at 50, 100, or 150 μg/kg/day, respectively, starting one week prior to epilepsy model induction. Meanwhile, sham and model mice received intraperitoneal injections of an equal volume of vehicle. Subsequently, KA was injected into the right hippocampus of both the model and treatment groups to induce seizures, whereas sham mice received an equal volume of saline administered in the same manner. The treatment group exhibiting the most favorable seizure latency was identified as the optimal‐dose group and used for subsequent experiments.

### 2.6 Kainic Acid (KA)‐Induced Epilepsy Model

Mice were anesthetized with tribromoethanol and secured in a stereotaxic apparatus (RWD Life Science, Shenzhen, China). Using the bregma as a reference point, 0.3 μg/μL KA (MedChemExpress, USA; HY‐N2309) was injected into the right hippocampal CA3 region (anterior/posterior: −2.46 mm; medial/lateral: 2.75 mm; dorsal/ventral: −2.63 mm) via a 5 μL microsyringe over a 3 min period. Following injection, the needle was left in place for 5 min and then withdrawn slowly to minimize reflux. The scalp was subsequently sutured and disinfected. Sham group mice received an equal volume of physiological saline administered in the same manner. Seizure severity was assessed using the Racine scale, where stage IV is characterized by bilateral forelimb clonus accompanied by rearing, and stage V by generalized tonic–clonic seizures with body rearing, loss of balance, and falling or rolling due to repeated convulsions [27]. Seizure latency was defined as the time from completion of KA injection to the onset of stage IV or higher seizures. A successful epilepsy model was defined by the consecutive occurrence of three stage IV or higher seizures. Thirty minutes after seizure onset, brain tissues were collected from successfully kindled mice for analysis.

### 2.7 Western Blot(WB)

Hippocampal tissues were placed in centrifuge tubes, homogenized in RIPA lysis buffer (R0010; Solarbio, China) supplemented with PMSF (P0100; Solarbio) and phosphatase inhibitors (P1260; Solarbio), lysed on ice for 30 min, and centrifuged at 12,000 × g for 15 min to collect the supernatant containing total protein. Equal amounts of protein, as determined by concentration, were transferred to new centrifuge tubes, mixed with SDS‐PAGE sample loading buffer (WB 2001; NCM Biotech, United States), and denatured by boiling. Proteins were separated by SDS‐PAGE and transferred onto PVDF membranes using a wet transfer system. Membranes were blocked in 5% nonfat milk (10 mM Tris, pH 7.6; 150 mM NaCl) for 1 h and incubated overnight at 4°C with primary antibodies: rabbit anti‐NCX1 (CY8139, Abways), rabbit anti‐GFAP (CY5424, Abways), rabbit anti‐NeuN (CY5515, Abways), and rabbit anti‐GAPDH (ab0037, Abways, China). Membranes were then incubated for 1 h at room temperature with the goat anti‐rabbit IgG (H^+^L) secondary antibody (1:10,000; SA 535571, Thermo Fisher Scientific, United States). Protein bands were visualized using a Li‐COR Odyssey Clx imaging system, and band intensities were quantified with ImageJ software.

### 2.8 Real‐Time Quantitative PCR (RT‐PCR)

Total RNA was extracted from hippocampal tissues using the NcmSpin Cell/Tissue Total RNA Kit (NCM Biotech, China), and RNA purity was assessed. RNA was reverse‐transcribed into cDNA using the Hifair AdvanceFast One‐Step RT‐gDNA Digestion SuperMix for qPCR kit (Yeasen Biotechnology, China) according to the manufacturer's instructions. RT‐PCR was performed on the Gentier 96E/96R PCR system (TIANLONG, China) using the Hieff UNICON Universal Blue qPCR SYBR Green Master Mix kit (Yeasen Biotechnology, China). Relative *SLC8A1* mRNA expression was quantified using the 2^−ΔΔCT^ method. PCR amplification was conducted with the following primers (5′–3′): *SLC8A1*, GAGCAGCCTTCAGAGCTGGTCGG (forward primer) and GTACAATAAGACTTCCAACTGC (reverse primer). Transcript levels of the target gene were normalized to *GAPDH*.

### 2.9 Immunofluorescence

Mouse brains were dehydrated, trimmed, embedded, and sectioned. Sections were blocked with 10% normal goat serum (Boster Biological Technology, Wuhan, China) at room temperature for 30 min. Primary antibodies (rabbit anti‐NCX1, DF9932, Affinity Biosciences; rabbit anti‐GFAP, CY5424, Abways; rabbit anti‐NeuN, CY5515, Abways) were applied and incubated overnight at 4°C. After washing with PBS (Zhongshan Golden Bridge Biotechnology, Beijing, China), sections were incubated with the appropriate secondary antibody (Servicebio, China) at 37°C for 1 h in the dark. Nuclei were counterstained with DAPI (Zhongshan Golden Bridge Biotechnology, Beijing, China) at room temperature for 10 min. Sections were rinsed with PBS and mounted with antifade mounting medium (Boster Biological Technology, Wuhan, China). Finally, high‐resolution Z‐stack imaging of the immunofluorescence‐stained sections was conducted using a confocal laser scanning microscope (Nikon AX), and orthogonal views (XY, XZ, and YZ planes) were reconstructed. Co‐localization was confirmed when fluorescent signals from two channels (e.g., NCX1 and GFAP) overlapped across all three orthogonal planes.

### 2.10 Flow Cytometry

Fresh hippocampal tissues were collected, washed, and then digested with trypsin. The digestion was terminated by adding DMEM containing 20% FBS, and the cell pellet was collected. The cell pellet was resuspended in the dark with the prepared calcium ion probe Fluo‐3 AM (IF0150, Solarbio, China). Staining was performed according to the manufacturer's instructions. Fluorescence intensity was measured using a flow cytometer (NovoCyte Advanteon, Agilent, China). The measurement value for each sample was the average fluorescence intensity of 10,000 cells.

### 2.11 Enzyme‐Linked Immunosorbent Assay (ELISA)

According to the manufacturer's instructions, glutamate (Glu) levels in mouse hippocampal tissues were quantified using a competitive inhibition ELISA kit (CES122Ge, Cloud‐Clone Corp., Wuhan, China). Optical density (OD) was measured at 450 nm using a microplate reader. A standard curve was generated from the OD values of the standards, and Glu concentrations in the samples were calculated accordingly.

### 2.12 H&E Staining and Nissl Staining

Mouse brain tissues were fixed, dehydrated through graded alcohols, cleared, paraffin‐infiltrated, embedded, and sectioned at a thickness of 4 μm. Sections were deparaffinized, rehydrated, stained, dehydrated, and mounted to prepare H&E and Nissl‐stained slides. Cellular morphology in hippocampal sections was examined using a light microscope (Olympus BX53, Tokyo, Japan). Images from the CA1 and CA3 regions were acquired, and the number of surviving neurons was quantified.

### 2.13 Statistical Analysis

Statistical analyses were performed using SPSS version 25.0 (IBM, Armonk, NY, USA). Descriptive statistics were calculated for all continuous variables, and data normality was assessed using the Shapiro–Wilk test. Normally distributed continuous variables are presented as mean ± standard deviation (SD). Comparisons between two groups were conducted using the independent samples *t*‐test. Additional statistical analyses and graphical presentations were generated using GraphPad Prism version 8.0.2 (GraphPad Software, San Diego, CA, USA). A two‐tailed *p*‐value < 0.05 was considered statistically significant.

## 3 Results

### 3.1 Ast_SLC8A1 Subpopulation May Mediate Epilepsy by Regulating the Calcium Signaling Pathway

**FIGURE 1:**
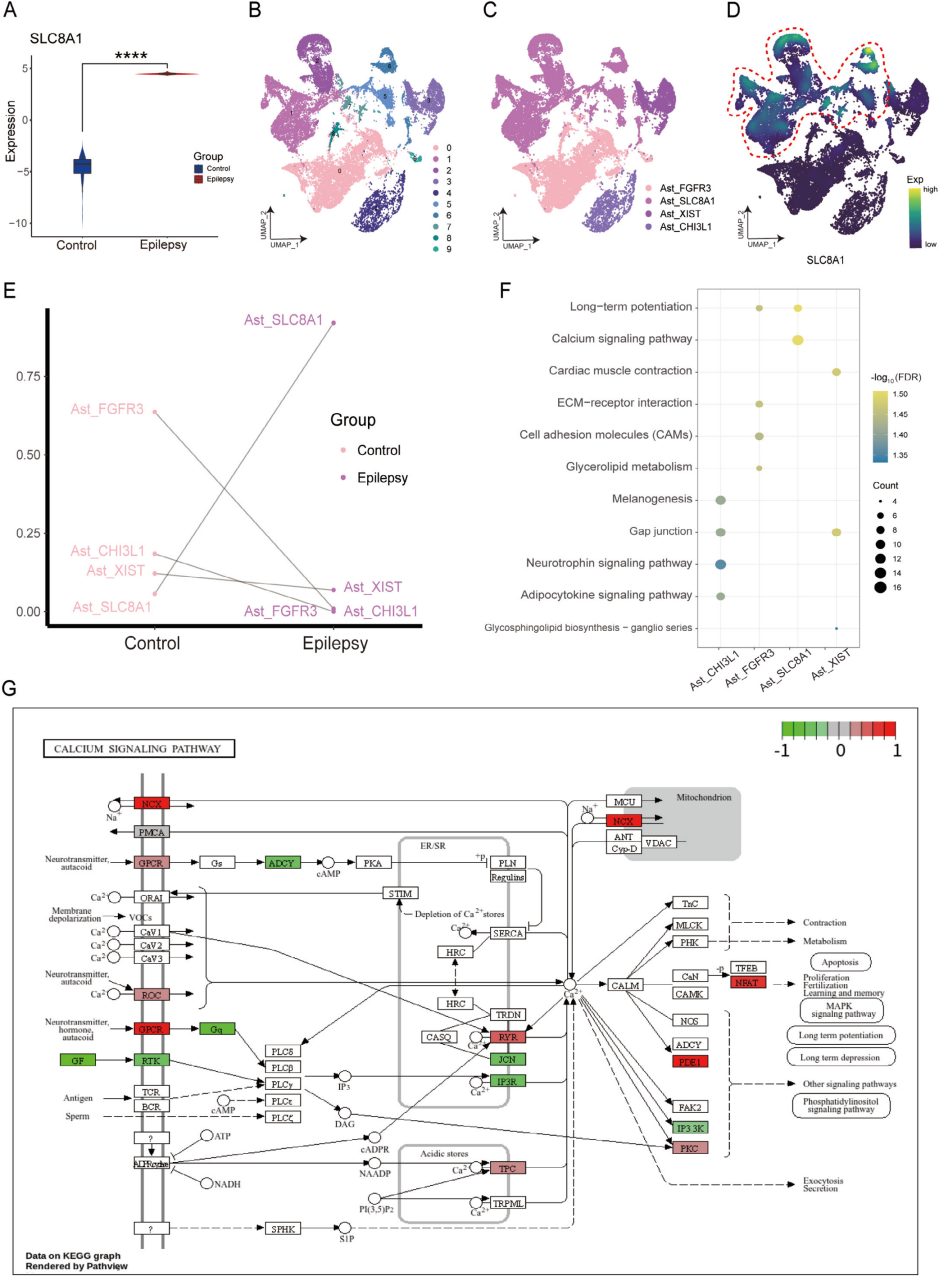
Role of astrocyte subpopulations in epilepsy. (A) Violin plot showing a significant upregulation of SLC8A1 mRNA expression in the epilepsy group compared with controls, ****p < 0.0001. (B, C) Single‐cell transcriptomic atlas of astrocyte subpopulations. (D) Density map illustrating the expression of the marker gene *SLC8A1* across astrocyte subpopulations. (E) Point line plots demonstrating differences in the proportion of astrocyte subpopulations between epilepsy and control brains. (F) Clustering‐bubble chart displaying biological functions and pathways significantly enriched in the astrocyte subpopulations. (G) Pathway map demonstrating the activation of the calcium signal pathway in the Ast_SLC8A1 subpopulation. Ast, Astrocyte.

Our bulk RNA‐seq analysis showed that in the epilepsy group, SLC8A1 mRNA showed a significant upregulation compared to the controls (Figure 1A). Sc/sn RNA‐seq data were collected from the GEO database, which included 25 epilepsy patients and 32 healthy controls. Stringent preprocessing and quality control resulted in a set of high‐quality single cells, with approximately 387,747 cells. When we did the clustering, 59 distinct populations appeared. We first used established marker genes to map seven major cell types and then analyzed the astrocytes (Ast) at a single‐cell resolution. The results showed that these cells were further clustered and subclustered into 10 initial groups (Figure 1B). From the above, we identified six distinct astrocytic subpopulations, each uniquely identified by specific molecular markers (as shown in Figure 1C), including the Ast_SLC8A1 subpopulation (as depicted in Figure 1D). Compared to controls, the Ast_SLC8A1 subpopulation showed a significant enrichment in the epilepsy group, which was a major component (Figure 1E). Ast_SLC8A1 was significantly enriched in calcium signaling and long‐term potentiation pathways, which was revealed by the enrichment analysis of astrocytic subpopulations (Figure 1F). KEGG pathway analysis further shows that Ast_SLC8A1 is linked to upregulation of key calcium signaling‐related genes like NCX (Figure 1G). These results indicate that the Ast_SLC8A1 subpopulation might play a role in the development of epilepsy by modulating calcium signaling pathways.

### 3.2 Docking Analysis of the Active Compound MCC‐135 Targeting NCX1

**FIGURE 2:**
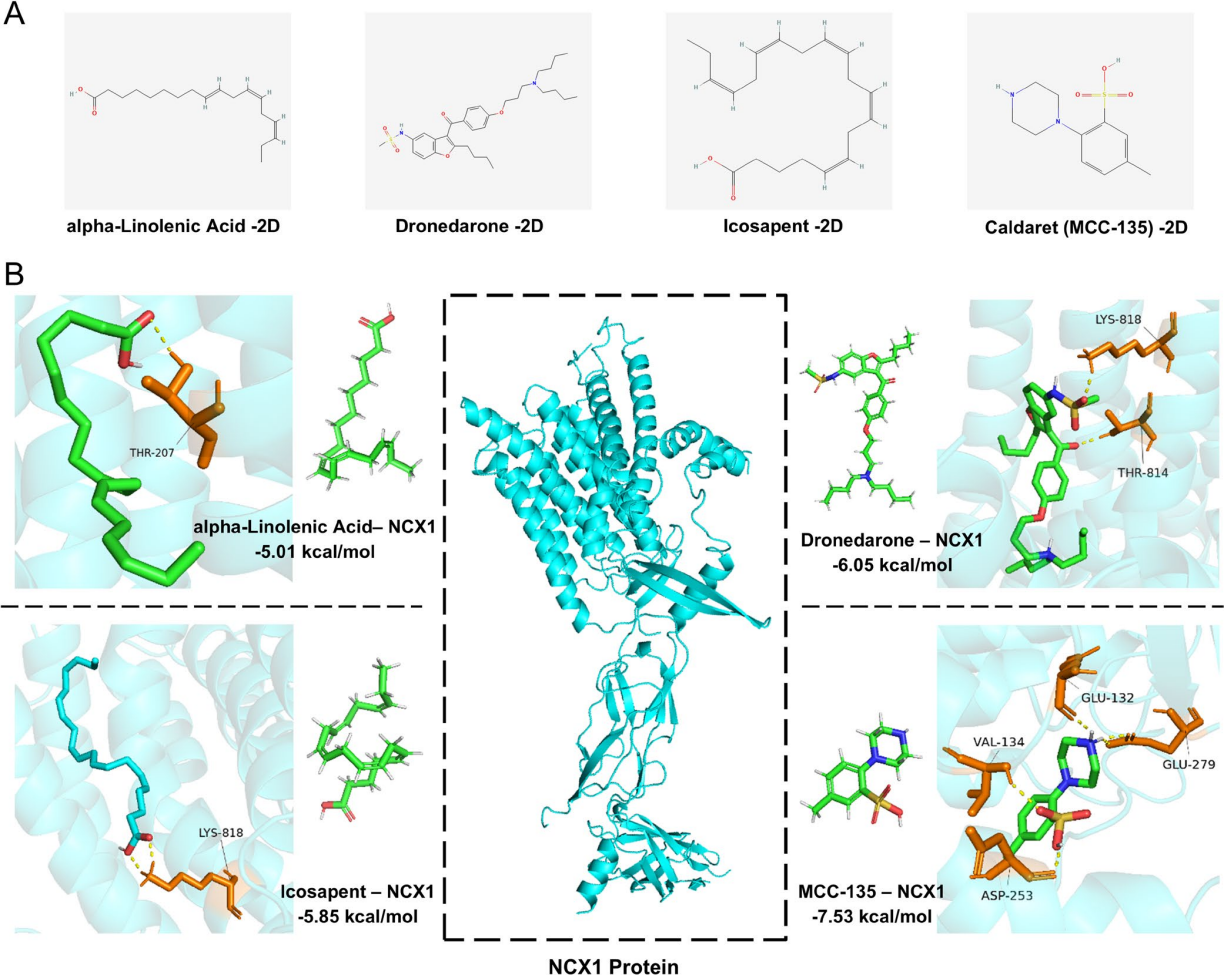
Molecular docking analysis targeting NCX1. (A) Two‐dimensional (2D) structures of small molecules. (B) Molecular docking models of NCX1 with multiple small‐molecule compounds.

We next retrieved active small molecules predicted to target the *SLC8A1* gene from the GeneCards database, including alpha‐Linolenic Acid, Icosapent, Dronedarone, and Caldaret (MCC‐135) (Figure 2A). The *SLC8A1* gene belongs to the solute carrier family 8 and encodes the NCX1 protein [28]. To evaluate potential interactions, molecular docking models were constructed using AutoDock for the NCX1 protein with alpha‐Linolenic Acid, Icosapent, Dronedarone, and MCC‐135. Their binding free energies were −5.01 kcal/mol, −5.85 kcal/mol, −6.05 kcal/mol, and −7.53 kcal/mol, respectively, indicating strong binding affinities with NCX1 (Figure 2B). Among these, MCC‐135 exhibited the strongest binding affinity with NCX1, primarily interacting with residues VAL‐134, GLU‐132, GLU‐297, and ASP‐253.

These findings suggest that MCC‐135, as an active compound, may exert therapeutic potential in epilepsy by regulating NCX1, thereby attenuating calcium signaling.

### 3.3 NCX1 Upregulation and co‐Localization With Astrocytes and Neurons in KA‐Induced Epileptic Mice

**FIGURE 3:**
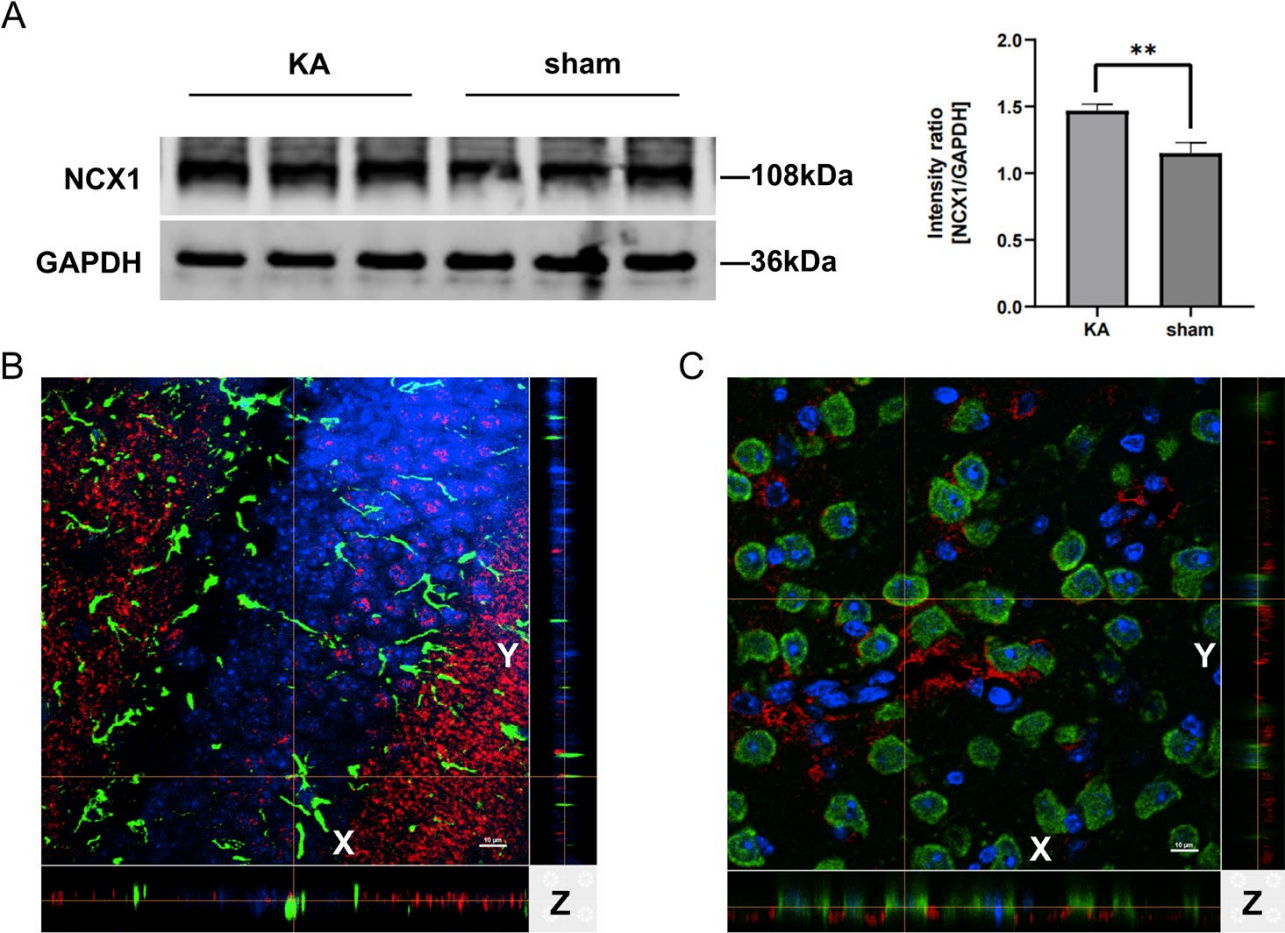
NCX1 upregulation and co‐localization with astrocytes and neurons in KA‐induced epilepsy. (A) WB and densitometry demonstrating significantly increased NCX1 expression in the hippocampus of KA‐induced epileptic mice, **p < 0.01. (B, C) Orthogonal view of immunofluorescence staining showing NCX1 (red) co‐localized with GFAP‐positive astrocytes (green, B) and NeuN‐positive neurons (green, C) in the mouse hippocampus. The stack of confocal sections was projected in three orthogonal planes (XY, XZ, YZ) at the level of the lines. Abbreviations: WB: Western Blot.

To experimentally validate the bioinformatics predictions, we successfully established a mouse model of epilepsy induced by kainic acid (KA). No seizures were observed in any Sham‐operated animals. WB analysis revealed that hippocampal NCX1 expression was significantly higher in the KA group compared with the sham group (Figure 3A,p < 0.01). Immunofluorescence staining was performed to examine the localization of NCX1 in relation to the astrocyte marker GFAP and the neuronal marker NeuN. Orthogonal views reconstructed from a confocal laser scanning microscope unequivocally demonstrated the co‐localization of NCX1 with both GFAP (Figure 3B) and NeuN (Figure 3C) in the mouse hippocampus.

### 3.4 MCC‐135 Improves Seizures in the KA‐Induced Epilepsy Mouse Model

**FIGURE 4:**
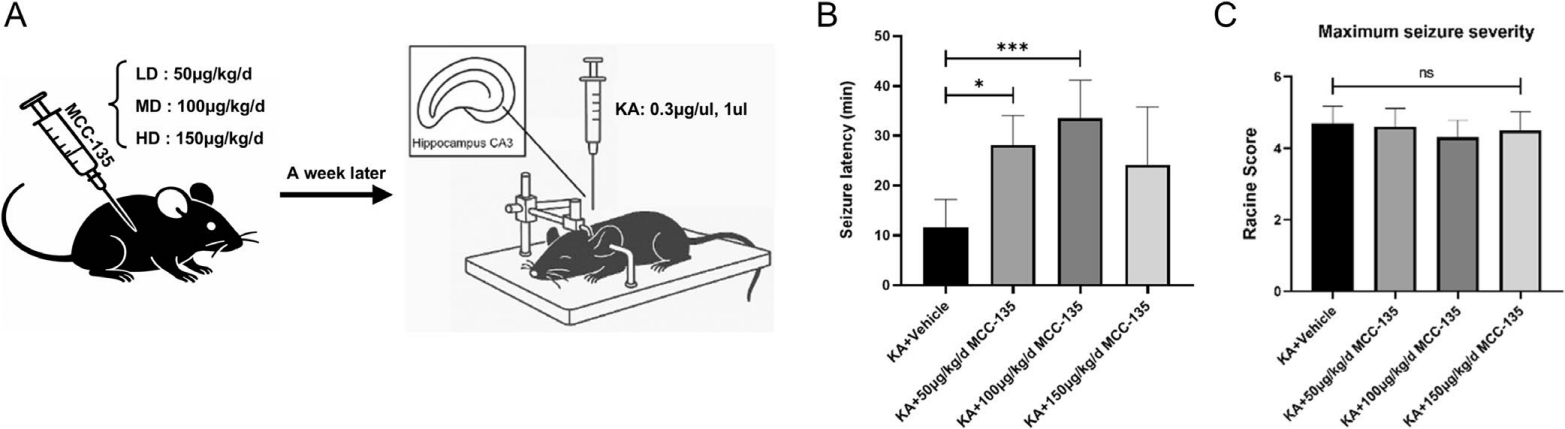
Anticonvulsant effects of MCC‐135 in KA‐induced epileptic mice. (A) Experimental design showing MCC‐135 administration and establishment of the KA‐induced epilepsy model. (B) Seizure latency in the KA‐induced epileptic mice was significantly prolonged by MCC‐135 treatment. *p < 0.05, ***p < 0.001. (C) Compared with the KA+ vehicle group, the score of seizure severity in the MCC‐135 treatment group showed a decreasing trend. *n* = 6 mice/group.

Prior to establishing the KA‐induced epilepsy model, mice were pretreated with MCC‐135 at three different doses (Figure 4A). Compared with the KA^+^ vehicle group, MCC‐135 treatment significantly prolonged the latency to KA‐induced seizure onset (min) (Figure 4B,p < 0.05 or *p* < 0.001). In addition, we observed a decreasing trend of the seizure severity score in the MCC‐135 treatment group (especially in the dose group of 100 μg/kg/d, *p* = 0.08) (Figure 4C). These findings indicate that MCC‐135 exerts anticonvulsant effects and warrants further investigation.

### 3.5 MCC‐135 Downregulates NCX1 Expression in KA‐Induced Epileptic Mice

**FIGURE 5:**
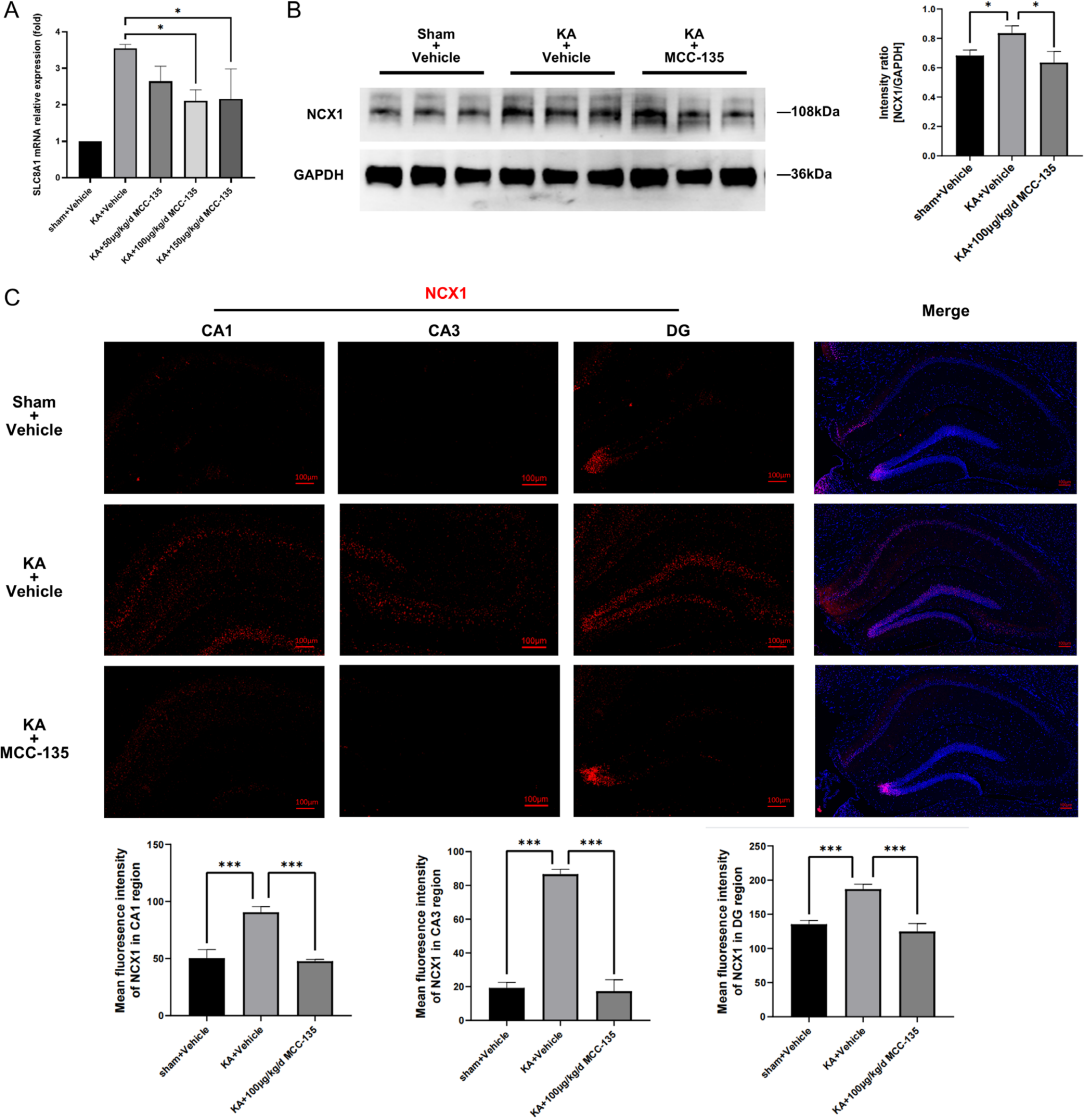
MCC‐135 Downregulates NCX1 Expression in KA‐Induced Epileptic Mice. (A) RT‐PCR analysis showed that compared with the KA group, *SLC8A1* mRNA levels were significantly reduced in the MCC‐135‐treated group, especially in the 100 μg/kg/d. (B) WB analysis indicated that MCC‐135 treatment markedly reduced NCX1 protein expression in the hippocampus of KA‐induced epileptic mice. (C) Immunofluorescence staining and quantitative analysis showed that NCX1 protein was significantly up‐regulated in CA1, CA3, and DG of the epileptic group, while it was significantly down‐regulated after MCC‐135 treatment. *p < 0.05，***p < 0.001. RT‐PCR, Real‐time quantitative PCR; WB, Western Blot.

RT‐PCR analysis showed that compared with the KA group, *SLC8A1* mRNA levels were significantly reduced in the MCC‐135‐treated group, especially in the 100 μg/kg/d (Figure 5A). The optimal dose was determined to be 100 μg/kg/d, which was selected for subsequent experiments. WB analysis demonstrated that NCX1 protein levels in the hippocampus of KA‐induced epileptic mice were markedly decreased after MCC‐135 treatment, returning to levels comparable with those of the control group (Figure 5B). Immunofluorescence staining and quantitative analysis of hippocampal sections further confirmed that NCX1 protein was significantly up‐regulated in CA1, CA3, and DG of the epileptic group, while it was significantly down‐regulated after MCC‐135 treatment (Figure 5C). These findings indicate that MCC‐135 exerts its effects primarily by downregulating NCX1 expression in the hippocampus of KA‐induced epileptic mice.

### 3.6 MCC‐135 Attenuates Intracellular Calcium Overload and Reduces Glutamate Levels in the Hippocampus of KA‐Induced Epileptic Mice

**FIGURE 6:**
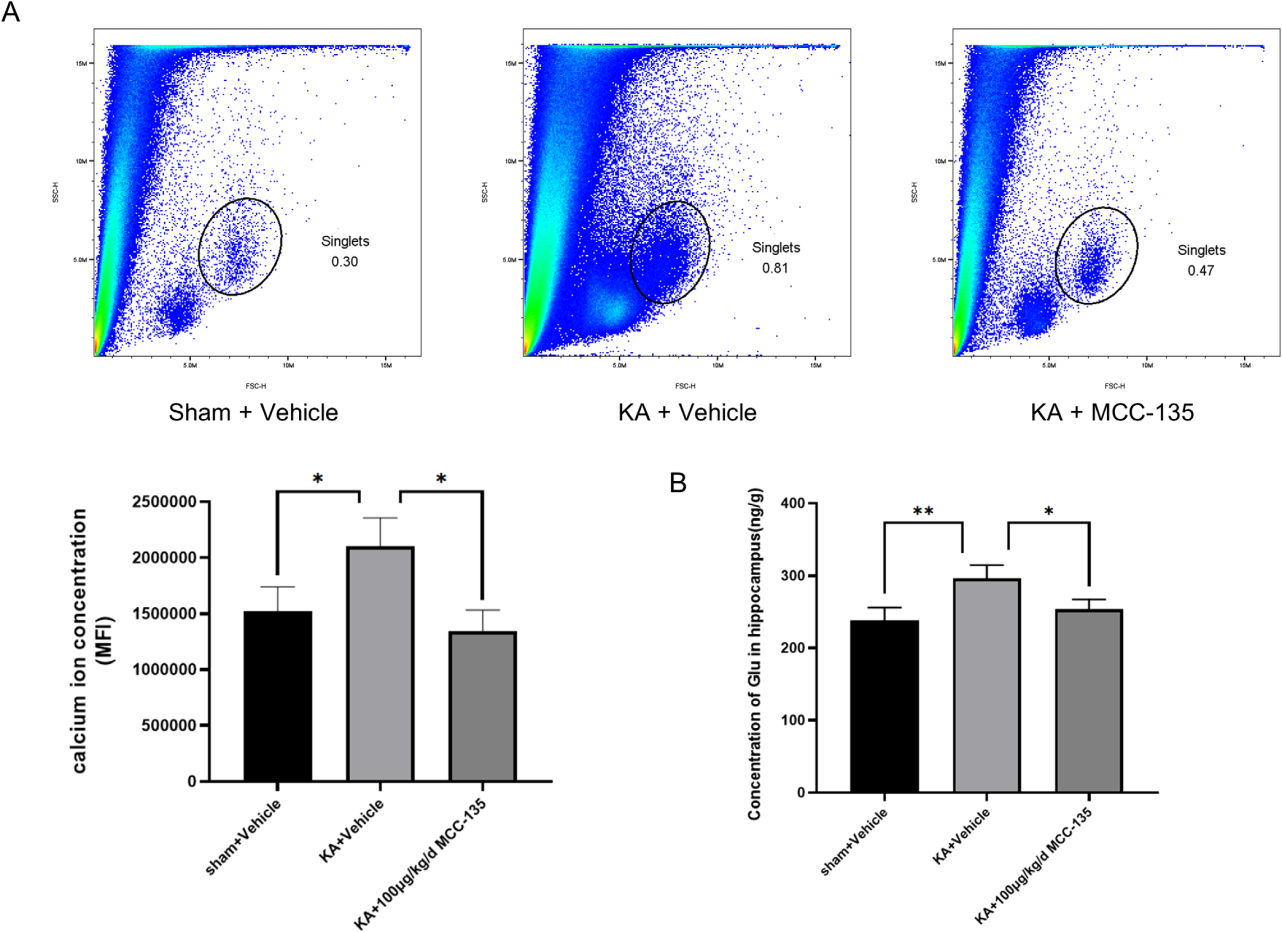
MCC‐135 Attenuates Intracellular Calcium Overload and Reduces Glutamate Levels in the Hippocampus of Epileptic Mice. (A) Flow cytometry analysis showed that the MFI of intracellular calcium in hippocampal cells was significantly reduced in MCC‐135–treated epileptic mice compared with the KA group. (B) ELISA results indicated that Glu levels were markedly elevated in the KA group, whereas MCC‐135 treatment significantly reduced hippocampal Glu concentrations. *p < 0.05, **p < 0.01. MFI, Mean fluorescence intensity; ELISA, Enzyme‐Linked Immunosorbent Assay; Glu, Glutamate.

Flow cytometry was performed to assess the mean fluorescence intensity (MFI) of intracellular calcium in hippocampal cells. Intracellular calcium levels were significantly elevated in KA‐induced epileptic mice, whereas MCC‐135 treatment markedly reduced calcium to levels comparable with the sham group (Figure 6A), indicating that MCC‐135 effectively alleviated calcium overload. ELISA analysis showed that Glu levels were significantly increased in the hippocampus of KA‐induced epileptic mice, whereas MCC‐135 treatment markedly reduced Glu concentrations (Figure 6B).

In summary, MCC‐135 suppressed NCX1 expression, alleviated hippocampal intracellular calcium overload, reduced Glu levels, and improved seizure outcomes, suggesting its therapeutic potential as an anticonvulsant agent.

### 3.7 MCC‐135 Exerts Neuroprotective Effects in KA‐Induced Epileptic Mice

**FIGURE 7:**
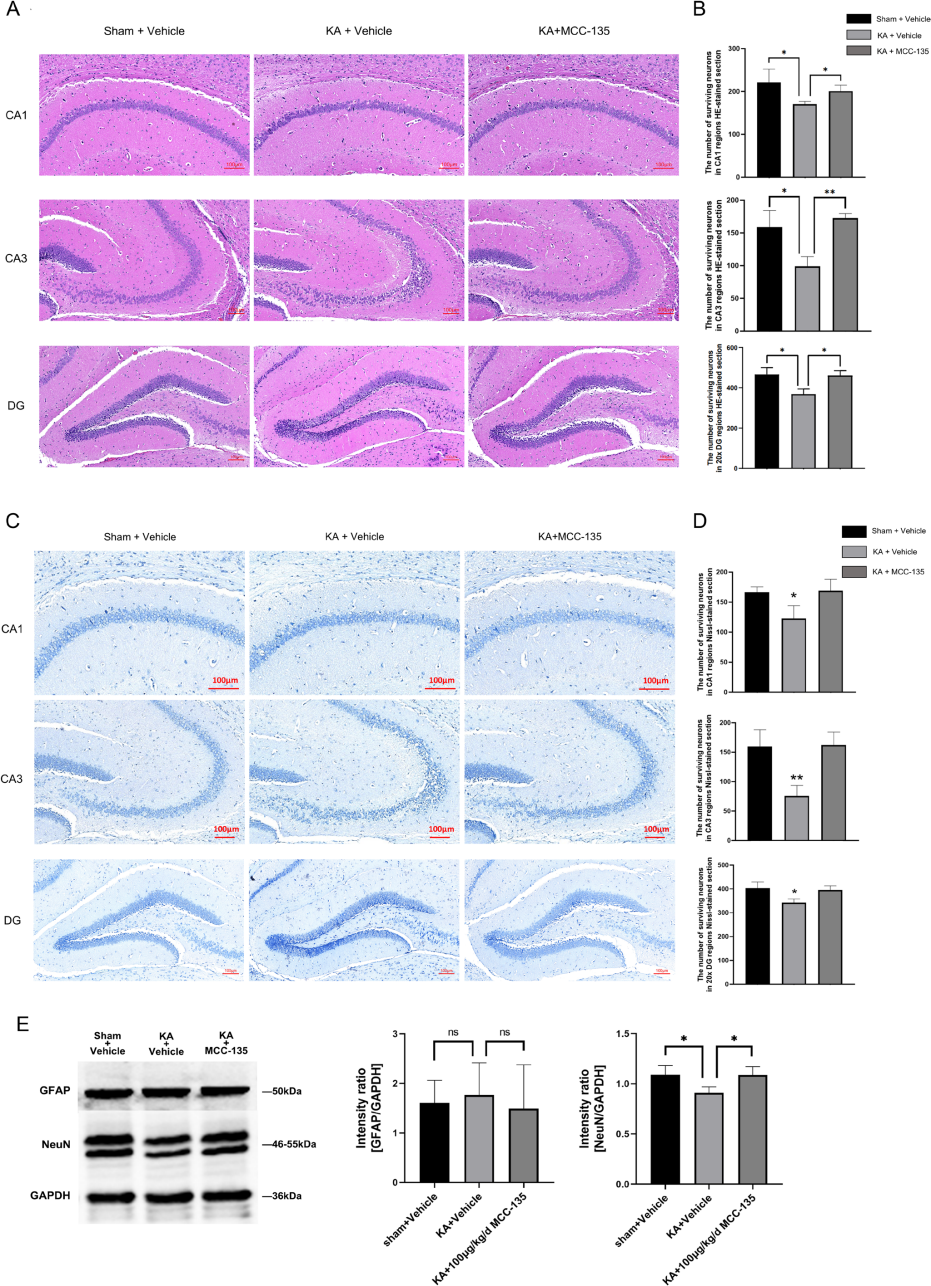
Neuroprotective Effects of MCC‐135 in KA‐Induced Epileptic Mice. (A, C) H&E and Nissl staining revealed marked neuronal damage in the hippocampal CA1, CA3, and DG regions of KA‐induced epileptic mice, which was substantially alleviated following MCC‐135 treatment. (B, D) Quantitative analysis of surviving neurons in the hippocampal CA1, CA3, and DG regions across groups. (E) WB analysis indicated that MCC‐135 treatment significantly increased NeuN protein expression in the hippocampus of KA‐induced epileptic mice. *p < 0.05, **p < 0.01.

H&E and Nissl staining revealed pronounced neuronal damage in the hippocampus of the KA^+^Vehicle group, characterized by nuclear pyknosis, rupture, lysis, and disorganized cell arrangement, particularly in the CA3 region. In contrast, these pathological alterations were alleviated in the hippocampus of KA‐induced epileptic mice treated with MCC‐135, with significantly fewer damaged neurons observed in the CA1, CA3, and DG regions (Figure 7A,C). Quantitative analysis demonstrated a significant increase in the number of surviving neurons in the hippocampal region of MCC‐135–treated mice compared with the KA model group (Figure 7B,D; *p* < 0.01 or *p* < 0.05). WB analysis indicated that MCC‐135 treatment significantly increased NeuN protein expression in the hippocampus of KA‐induced epileptic mice (Figure 7E; *p* < 0.05), whereas no significant change in GFAP expression was observed across groups.

These findings indicate that MCC‐135 mitigates neuronal damage in KA‐induced epileptic mice, exerting neuroprotective effects.

## 4 Discussion

Epileptogenesis is described as a result of the imbalance between excitation and inhibition within neuronal networks, leading to excessive and hypersynchronous neuronal activity that disrupts normal brain function [29]. Currently, the most commonly used approach to treat epilepsy is pharmacotherapy with ASMs. Although approximately 70% of patients can achieve seizure‐free status with these drugs, about 30% of patients develop drug‐resistant epilepsy (DRE) [30]. While a substantial proportion of these patients may achieve seizure freedom through epilepsy surgery or other non‐pharmacological treatments (e.g., dietary therapy and neurostimulation), most endure years of intractable and disabling seizures. DRE imposes a significant economic burden on individuals, their families, healthcare systems, and society, including the direct physical and psychosocial impact of seizures, as well as the burden of comorbid mental, cognitive, and physical disorders [31]. To improve seizure control rates and enhance the quality of life for epilepsy patients, we are committed to identifying potential drug targets and developing new ASMs.

Our single‐cell transcriptomic analysis revealed that the Ast_SLC8A1 subpopulation is significantly enriched in epileptic brains and strongly implicated in the regulation of calcium signaling pathways. Molecular docking analysis further indicated that the bioactive small molecule MCC‐135 exhibits strong binding affinity to NCX1, which is encoded by *SLC8A1*. Based on these bioinformatics predictions, we validated the molecular alterations and systematically evaluated the therapeutic efficacy and underlying mechanisms of MCC‐135 in a KA‐induced mouse model of epilepsy.

This study indicates that NCX1 levels are significantly upregulated in the KA‐induced acute epilepsy mouse model. Notably, previous studies have provided conflicting evidence regarding the changes in NCX1 levels in epilepsy. Studies have shown that NCX1 knockout mice are resistant to PTZ‐induced seizures, and inhibitors of NCX1 reverse mode can reduce both the incidence and severity of seizures in animals [32]. Furthermore, NCX1 levels are significantly increased in the brain tissue of neonatal rats following Glu induction [33]. However, in some chronic epilepsy animal models, NCX1 levels are downregulated [34, 35]. On one hand, this could be related to the heterogeneity of NCX1 transport activity across different epilepsy models, and on the other hand, it may be associated with neuronal loss in chronic epilepsy models. Given that both pharmacological inhibition of NCX1 reverse function and downregulation of NCX1 expression have anticonvulsant effects, this suggests that the reverse mode of NCX1 contributes to the occurrence of epilepsy [36].

Our orthogonal views of immunofluorescence staining revealed co‐localization of NCX1 with both astrocytes and neurons in the mouse hippocampus. NCX1 on astrocytes mediates Ca^2+^ transport and regulates intracellular Ca^2+^ homeostasis in these glial cells [8]. Astrocytes modulate neuronal and synaptic activity by releasing neurotransmitters (e.g., Glu, D‐serine, GABA) in a Ca^2+^‐dependent manner [37]. A wealth of experimental evidence from various brain regions (both cultured and acutely isolated tissue slices) has demonstrated that neuronal activity, exogenous Glu application, or activation of ionotropic Glu receptors and Glu transporters transiently elevates Na^+^ levels in astrocytes. This transient Na^+^ influx triggers reverse‐mode operation of NCX, thereby promoting Ca^2+^ influx and generating astrocytic Ca^2+^ signaling cascades [38]. The Ca^2+^ influx via reverse‐mode NCX is a key driver of Glu release from astrocytes, which is further enhanced by depolarization [39]. Released Glu can activate postsynaptic NMDA receptors (NMDARs), inducing slow inward currents (SICs) that synchronize hippocampal pyramidal neurons. This increase in neuronal excitability represents a non‐synaptic mechanism of neuronal synchronization, which is implicated in the pathogenesis of neurological disorders such as epilepsy, stroke, and neurodevelopmental diseases [40]. In our study, the levels of NCX1, intracellular Ca^2+^, and Glu were significantly elevated in the hippocampus of KA‐induced epileptic mice. These findings suggest that reverse‐mode NCX1 may mediate epileptogenesis by regulating astrocytic Ca^2+^ levels and promoting Glu release. Thus, NCX1 represents a potential therapeutic target for epilepsy.

Previously mentioned, MCC‐135 reduces intracellular Ca^2+^ overload by downregulating NCX expression in the plasma membrane or by enhancing sarcoplasmic reticulum Ca^2+^ uptake capacity [11]. To date, research on MCC‐135 has mainly focused on its cardioprotective effects in myocardial ischemia and infarction [41]. Previous studies have shown that the cardioprotective role of this substance during ischemia/reperfusion injury is directly linked to the reduction of calcium overload [42]. However, the role and underlying mechanisms of MCC‐135 in epilepsy remain largely unexplored. In this study, we first demonstrate that MCC‐135 suppresses NCX1 expression, alleviates intracellular Ca^2+^ overload, and reduces Glu levels in the hippocampus of KA‐induced epileptic mice, which is linked to a significant improvement in seizure activity. Moreover, the seizure severity scores in the MCC‐135 group showed a decreasing trend. This reduction didn't reach statistical significance, but it matches the beneficial effects seen for other endpoints, like prolonged seizure latency, neuroprotection, and molecular marker changes. These findings indicate that MCC‐135 might have the potential to reduce seizure severity. MCC‐135 attenuates Ca^2+^ overload and reduces excitatory neurotransmitter levels, offering a new pharmacological viewpoint on epilepsy treatment, broadening its potential therapeutic applications beyond cardiovascular contexts.

The most common pathology observed in temporal lobe epilepsy (TLE) patients is hippocampal sclerosis, characterized by loss of pyramidal cells in the CA regions of the hippocampus, gliosis, granule cell dispersion, and axonal sprouting [43]. In TLE patients, neurons in the CA regions are particularly vulnerable to damage, with losses ranging from more than 50% to nearly complete neuronal loss [44]. In this study, MCC‐135 treatment significantly reduced neuronal damage in the CA1, CA3 and DG regions of the hippocampus in epileptic mice. Furthermore, this study indicates that there is no significant difference in GFAP protein expression among the groups, which is an interesting observation. We speculate that this might be related to the time point of the analysis. Previous studies have shown that the proliferation of astrocytes occurs three days after status epilepticus [45], a time point following the protein analysis in this study. Nevertheless, we observed that the MCC‐135 group seemed to have a tendency to suppress astrocyte activation, which requires further targeted investigation in the future. These findings suggest that MCC‐135 exerts a neuroprotective effect, preserving hippocampal neurons and potentially mitigating hippocampal sclerosis in epilepsy.

All in all, our findings strongly suggest that MCC‐135 exerts therapeutic effects in epilepsy by downregulating NCX1 expression, thereby reducing intracellular Ca^2+^ overload and glutamate levels. Moreover, MCC‐135 treatment greatly reduced the neuronal damage in the hippocampal CA1, CA3, and DG regions, indicating a neuroprotective effect. These results fill an important gap in the current understanding of MCC‐135's role in both antiepileptic therapy and neuronal protection and provide compelling evidence to support its potential as a novel candidate for epilepsy treatment.

However, this study has several limitations. Firstly, this study was unable to directly confirm the pharmacological dependence of MCC‐135 on NCX1 through genetic approaches such as knockdown or knockout. Future validation using conditional NCX1 knockout models will be essential to conclusively establish NCX1 as a key target of MCC‐135. Secondly, the molecular docking analysis performed here relied on a representative NCX1 structure. Given the presence of multiple alternative splicing variants of SLC8A1, further comparative studies on the interaction between MCC‐135 and different NCX1 subtypes will help clarify its binding specificity and pharmacological mechanisms. Additionally, in order to unify the duration of seizures among the groups, the tissue was collected at a fixed point of 30 min after seizure onset in mice, which precluded the recording of total seizure duration. This aspect will be addressed in future experimental designs. It is worth noting that in the KA‐induced acute epilepsy model, the criterion for successful induction is the occurrence of three consecutive grade 4–5 seizures. Therefore, it is challenging in itself to observe a significant change in the maximum seizure severity in this model. This difference between groups may become more evident with larger sample sizes or in chronic epilepsy models. These efforts will further clarify the therapeutic potential of MCC‐135 and contribute to clinical transformation.

## 5 Conclusion

This study demonstrates that MCC‐135 exerts both neuroprotective and anticonvulsant effects in epilepsy by downregulating NCX1 expression, thereby alleviating intracellular Ca^2+^ overload and reducing glutamate levels in the hippocampus. To our knowledge, this is the first study to propose that MCC‐135 reduces seizures by downregulating NCX1 expression to decrease intracellular calcium overload in the hippocampus. These findings fill a critical gap in the fields of antiepileptic therapy and neuronal protection and provide novel insights into the therapeutic potential of MCC‐135 as an antiepileptic agent.

## Author Contributions

Chaoning Liu: Conceptualization, methodology, formal analysis, validation, writing – review and editing, writing – original draft. Min He and Rida Li: Conceptualization, validation, software, writing – review and editing, writing – original draft. Shouhuan Zheng: Conceptualization, methodology, software, writing – original draft. Lanfeng Sun: Validation, visualization. Chi Gong: Validation. Hengchang Qi and Xinran Qin: Data curation. Xiaohang Gan and Fang Wang: Formal analysis. Yuan Wu: Funding acquisition, investigation, resources, supervision, writing – review and editing.

## Ethics Statement

The animal study was approved by the Animal Care & Welfare Committee of Guangxi Medical University (No.: 202504005). The study was conducted in accordance with the local legislation and institutional requirements.

## Disclosure

Generative AI Statement: The authors declare that no Generative AI was used in the creation of this manuscript.

## Conflicts of Interest

The authors declare no conflicts of interest.

## Data Availability

Data was downloaded from the public database online at the GEO database under accession numbers GSE160189, GSE221849, GSE241521, GSE129308, GSE157827, GSE174367, GSE178175, GSE213364, GSE140393, GSE163737, GSE33000, and GSE63808.
